# DIP–STR: Highly Sensitive Markers for the Analysis of Unbalanced Genomic Mixtures

**DOI:** 10.1002/humu.22280

**Published:** 2013-01-25

**Authors:** Vincent Castella, Joëlle Gervaix, Diana Hall

**Affiliations:** Unité de Génétique Forensique, Centre Universitaire Romand de Médecine Légale, Centre Hospitalier Universitaire Vaudois et Université de LausanneLausanne, 1011, Switzerland

**Keywords:** compound genetic marker, forensic, DNA microchimerism, diagnostics

## Abstract

Samples containing highly unbalanced DNA mixtures from two individuals commonly occur both in forensic mixed stains and in peripheral blood DNA microchimerism induced by pregnancy or following organ transplant. Because of PCR amplification bias, the genetic identification of a DNA that contributes trace amounts to a mixed sample represents a tremendous challenge. This means that standard genetic markers, namely microsatellites, also referred as short tandem repeats (STR), and single-nucleotide polymorphism (SNP) have limited power in addressing common questions of forensic and medical genetics. To address this issue, we developed a molecular marker, named DIP–STR that relies on pairing deletion–insertion polymorphisms (DIP) with STR. This novel analytical approach allows for the unambiguous genotyping of a minor component in the presence of a major component, where DIP–STR genotypes of the minor were successfully procured at ratios up to 1:1,000. The compound nature of this marker generates a high level of polymorphism that is suitable for identity testing. Here, we demonstrate the power of the DIP–STR approach on an initial set of nine markers surveyed in a Swiss population. Finally, we discuss the limitations and potential applications of our new system including preliminary tests on clinical samples and estimates of their performance on simulated DNA mixtures.

## Introduction

Genetic polymorphisms such as short tandem repeats (STR) and single-nucleotide polymorphism (SNP) are commonly used in forensic identity testing [Gill et al., 1994, 2001; Jobling and Gill, 2004; Kayser et al., 1997; Moretti et al., 2001; Tully et al., 2001] and medical genetics [Goate et al., 1991; Hastbacka et al., 1992; Houwen et al., 1994; Wooster et al., 1995]. However, because of PCR-based analytical methods, these markers have low sensitivity in characterizing samples that contain DNA from several contributors in very different proportions. In general, PCR fragment analysis that is not deep sequenced allows for the detection of a minor DNA component in a mixture only when it represents more than 10% of the total DNA; however, the unambiguous identification of all minor DNA alleles requires a minor DNA fraction of at least 20% [Fregeau et al., 2003; Sutherland et al., 2009; Westen et al., 2009].

Historically, characterizing unbalanced DNA mixtures has been associated with the analysis of biological stain for forensic identification purposes. For example, challenging mixed stains may be derived from samples including, but not limited to, clothing, hair, skin, or items the perpetrator may have touched. Because these samples are likely to carry small quantities of the perpetrator's DNA mixed with a large amount of the victim's DNA, the limit of resolution of currently used markers may have dramatic consequences for justice. Moreover, several fields of medical genetics recently expressed a paramount need for tools that enable the analysis of unbalanced DNA mixture occurring in vivo, also referred as DNA microchimerism (less than 1% of foreign cells). Few examples are the DNA microchimerism associated to pregnancy, which is caused by the transient circulation of minute quantities of fetal DNA in the maternal blood [Lo et al., 1998; Tjoa et al., 2006] or the trace quantities of donor's DNA in the body fluids (blood and urine) of transplanted patients [Chok et al., 2002; Gadi et al., 2006; Moreira et al., 2009; Pujal and Gallardo, 2008].

Advances in the above medical fields all require novel analytical approaches for the detection and quantification (specific for organ transplant applications) of the DNA of interest when mixed to a high foreign DNA background. A simple solution to this problem is represented by a standard amplification-based method that targets a genomic region unique to the minor DNA (allogeneic marker) eliminating the masking effect of the major DNA.

Molecular markers located on the Y chromosome are the most widely employed allogeneic markers for all cases of male DNA detection over high female background [Roewer, 2009]. Unfortunately, this approach has several limitations: first, the applicability to mixtures with a specific sex mismatch dramatically reduces the number of suitable cases. Second, because of the mostly nonrecombining nature of the Y chromosome, without mutations paternally related individual all share the same Y STR alleles. Third, since all Y STR constitute a single haplotype, multiplying the individual allele frequencies is not valid as for independently inherited autosomal STR. Therefore, a match between Y STR profiles that is evaluated on the basis of haplotype frequencies may be an evidence of reduced weight for forensic identification purposes [Vermeulen et al., 2009]. On this issue, a sequencing-based study of two Y chromosomes separated by 13 generations discovered four single-base differences in 10 Mb DNA [Xue and Tyler-Smith, 2010]. This suggests that the Y chromosome accumulates around one mutation per generation, which means that a sequencing-based assay should distinguish almost every Y chromosome. Alternatively, it was recently proposed a locus-specific approach, which is based on the analysis of 13 rapidly mutating Y-STRs. Although this method can be more easily executed, the results indicate a lower performance with about 50% of father and sons being distinguished among 305 male relatives [Ballantyne et al., 2012].

The human leukocyte antigen (HLA) gene variants are another example of allogeneic markers, and are typically employed in transplantation follow-up studies [Gadi et al., 2006]. Nevertheless, this method uses a genetic marker that has an effect on the immunogenic compatibility of the recipient–donor pair; therefore, it necessarily introduces an ascertainment bias in the microchimerism analysis induced by organ transplant. Additionally, as for the Y chromosome, HLA variants are clustered on the same chromosome, resulting in a decreased power of discrimination between individuals.

In peripheral blood DNA macrochimerism (usually more than 10% of foreign cells) induced by hematopoietic stem cell transplantation, new assays have been proposed for all donor–recipient type based on biallelic deletion–insertion polymorphisms (DIP) polymorphisms and null alleles [Alizadeh et al., 2002; Jimenez-Velasco et al., 2005]. Yet, biallelic systems are associated with a low discrimination power which makes them sensitive to confounding factors such as transfusions associated to the surgery, inborn microchimerism [Rubocki et al., 2001] or other possible medically related contaminations linked to the amplification of minute quantities of DNA.

Finally, great expectations are placed on the contribution of next-generation sequencing techniques to forensic and DNA microchimerism analyses [Irwin et al., 2011; Snyder et al., 2011]. However, a recent publication indicates that caution must be taken [Bandelt and Salas, 2012]. These authors conducted an indirect quality assessment of a study on heteroplasmic mitochondrial DNA mutations based on high-throughput sequencing [He et al., 2010]. By using in silico phylogenetic approaches, they found that on average at least five mutations were missed per sample. Although this is one particular study and the error rate may dramatically reduce with the rigorous application of quality controls and standards to be defined, next-generation sequencing may remain of limited use for immediate forensic applications. Cautions are mainly about: the quantity and quality of DNA required; the persistence of PCR bias as forensic applications is expected to use target sequencing approaches (not whole genome); the risk of cross contamination is due to a large number of parallel reactions; the difficulty of repeat sequence analysis (STR) and finally; the times, costs, and expertise required for this type of analysis [Berglund et al., 2011; Hert et al., 2008; Metzker, 2010; Snyder et al., 2011].

With these considerations in mind, we propose here new genetic markers, the DIP–STR, which are located throughout the genome, highly polymorphic, easy to genotype, and capable of resolving extremely unbalanced two DNA mixtures (ratio 1:1,000). We describe an initial set of DIP–STR markers including a population survey and data of their performance on both simulated DNA mixtures and two representative clinical samples. Further, we discuss the potential contribution of this tool to various research fields.

## Methods

### Database Search of DIP-Linked STR Markers

From the University of California Santa Cruz (UCSC) database ([assembly Feb2009(GRCh37/hg19]) [Kent et al., 2002], by using the “Table Browser” tool, we searched for DIP polymorphisms in the group “Variation and Repeats,” track “SNPs (131)”, region “genome” filtered by class “in-del”, “insertion” and “deletion”; those with “unknown” validity were excluded and we set sequence “weight” equal to 1. About 216,000 DIP were obtained on the basis of these criteria, this number still included stretches of the same base and short nucleotide repeats, which we excluded after selection of DIP-linked STR. From the same database, STR were selected from the group “Mapping and Sequencing Tracks”, track “STS Markers” filtered for the presence on the “Marshfield genetic map” by selecting those with assigned “MarshfiledChrom” and “MarshfieldPos”, we further eliminated those with a Marshfield chromosome assignment not corresponding to the UCSC chromosome assignment. About 7,469 STR were selected on the basis of these criteria. The DIP characterized by a deletion/insertion of at least 2 bp located less than 500 bp from an STR were about 70. To ensure the independence of markers and to reduce the risk of linkage with disease or other phenotypic information, we searched for candidate DIP–STR located on different chromosomes (when possible) and in noncoding regions, which are not clinically associated. Once an initial list of nine DIP–STR markers was established (Table 1), we tested single-marker allelic variability by genotyping 20 Swiss individuals under informed consent.

**Table 1:** DIP–STR Marker List

DIP–STR	Chromosome	DIP S/L sequence	STR repeat	DIP–STR size (bp)
MID1013^a^–D5S490	5q23.2	–/CCAG	GT	307–343
MID1950^a^–D20S473	20p13	–/ATT	TTA	213–231
MID1107^a^–D5S1980	5p15.33	–/AACA	CA	650–680
rs11277790–D10S530	10q25.1	–/TCCAACT	GT	344–362
rs60194384–D15S1514	15q26.2	–/TCTTAA	TATC	281–309
rs67842608–D5S468	5q11.2	–/TGGTTTAA	GT	379–395
rs66679498–D2S342	2q32.3	–/CCAACTTTCTCCTAC	CA	340–357
rs10564579–D3S1282	3p24.1	–/GTCATA	CA	714–728
rs35708668–D5S2045	5q34	–/TACTATGTAC	CA	621–649

a Marker name from the Marshfield database correspond to rs1611095, rs2308142, and rs2067195, respectively.

### Primer Design and PCR Conditions

Each single DIP and STR polymorphism was first independently validated by PCR. To do so, we used the primer3 software [Rozen and Skaletsky, 2000] for designing primers around the DIP polymorphisms; we used the primers indicated on the UCSC database for the STR. The L-DIP primers were designed to include a 3′end region that was complementary to the inserted sequence, whereas the S-DIP primer lacked the inserted sequence at the 3′end and passed the insertion point by three to seven nucleotides (Supp. Table S1). It should be noted that a condition for strong specificity of both S and L primers is that the inserted sequence and the region past the insertion point are substantially different.

Each PCR reaction was performed in a final volume of 20 µl containing 1× PCR buffer with 1.5 mM MgCl_2_ (Applied Biosystems, Zug, Switzerland), 250 µM of each dNTP, 1.2 U AmpliTaq Gold DNA Polymerase, 1 µM of each forward and reverse primer and 1 ng of genomic DNA (DNA quantity varied for specificity and limit of detection tests). The PCR thermocycling conditions are: 5 min at 95°C; followed by 60 sec at 94°C, 60 sec at 52°C, and 60 sec at 72°C for 30 cycles and a final extension of 30 min at 72°C. The annealing temperature of 52°C was modified to 58°C when using DIP–STR primers MID1107-D5S1980 and both annealing and extension time were 75 and 90 sec for marker MID1107-D5S1980 and rs10564579-D3S1282 and rs35708668-D5S2045. The thermal cyclers employed are GeneAmp 9700 (Applied Biosystems, Zug, Switzerland). Before capillary electrophoresis, 1 µl PCR product is added to 8.5 µl deionized formamide HI-DI (Applied Biosystems, Zug, Switzerland) and 0.5 µl GS-ROX 500 size standard (Applied Biosystems, Zug, Switzerland). DNA fragments are separated using an ABI PRISM 3100 Genetic Analyzer (Applied Biosystems, Zug, Switzerland) according to manufacturer's instruction and analyzed with GeneMapper ID v3.2 software (Applied Biosystems, Zug, Switzerland), with minimum interpretation peak threshold of 50 relative fluorescence units (RFU).

### Allele Specificity Tests

**Figure 1:**
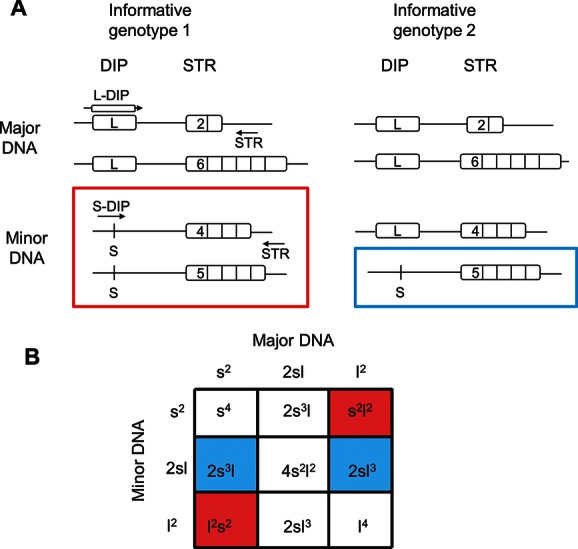
**A:** DIP–STR informative genotypes. A DIP–STR haplotype is analyzed by using PCR primers overlapping the DIP on one side (either L-DIP or S-DIP primer) and downstream the STR (STR primer) on the other side. When major and minor DNA contributors are opposite homozygous LL/SS or SS/LL (“informative genotype 1”), two minor DNA haplotypes can be identified in the mixture (red box); conversely, when the major DNA contributor is homozygous, either SS or LL, and the minor DNA contributor is heterozygous (“informative genotype 2”), one minor DNA haplotype can be identified in the mixture (blue box). Arrows indicate PCR primers. **B:** Theoretical evaluation of the occurrence of informative markers. Letter *s* and *l* indicate the allele frequencies of S and L alleles, respectively. In red are the probabilities of informative DIP genotypes enabling the identification of two minor DNA haplotypes (“informative genotype 1”). Depending on the linked STR alleles, these are the same or different. In blue are the probabilities of informative DIP genotypes enabling the identification of one minor DNA haplotype (“informative genotype 2”). The sum of these values (*s*^2^*l*^2^+*l*^2^*s*^2^+2*s*^3^*l*+2*sl*^3^) gives the probability of having any type of informative genotype (*I*) in a mixture of two DNA.

**Figure 2:**
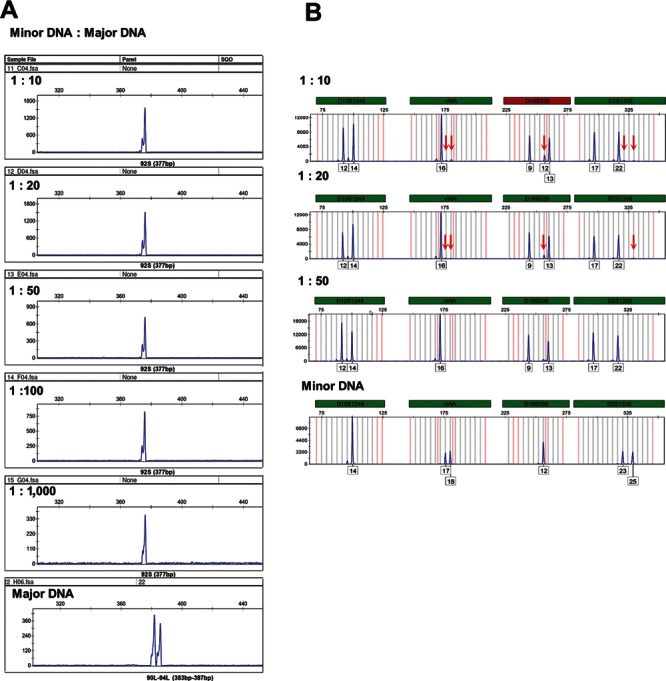
Performance of DIP–STR and forensic STR in resolving various ratios of DNA mixtures. **A:** The electropherograms show the DIP–STR-specific amplification of a minor DNA when mixed to increasing quantities (1:10 to 1:1,000) of a second DNA, both genotypes of the two-mixed DNA are known. We report here one informative marker, rs67842608-D5S468 as a representative example. The minor DNA-specific amplification is achieved by using the S-DIP primer, which targets the minor allele 92S (377 bp), that is not shared with the major DNA, 90L 94L (383–387 bp). Note that the alleles of the major DNA are 375 and 379 bp long, when they are amplified by the S-DIP primer due to a nonspecific amplification. **B:** The same DNA mixtures were analyzed by the forensic kit NGMselect (Applied Biosystems). We show here only four representative markers. The electropherogram corresponding to the DNA ratio 1:10 shows that the minor DNA alleles indicated by red arrows are close to the detection limit and they are quickly not detected anymore when the quantity of the major DNA increase to 1:20, 1:50, and so on (for 1:100 and 1:1,000 data are not reported).

Amplification specificity, when using S-DIP and STR primers as well as L-DIP and STR primers, was tested by amplifying 1 ng of DNA template heterozygous for the DIP allele mixed with increasing quantities of a second DNA homozygous for the opposite DIP allele LL and SS respectively (as “informative genotype 2” in Fig. 1A), both DNA were selected irrespective of the genotype of the linked STR. The DNA ratios tested included 1:10, 1:20, 1:50, 1:100, and 1:1,000. One representative result is reported in Figure 2 together with the comparative analysis of the same mixture amplified by a standard forensic kit, NGMselect (Applied Biosystems, Zug, Switzerland) according to manufacturer's instruction.

### PCR Limit of Detection

We estimated the minimal amount of DIP heterozygous DNA required to produce a DIP–STR product detectable by capillary electrophoresis, by varying the total DNA content in the PCR reaction from 1 down to 0.025 ng. Amplifications were done in duplicate, and the number of PCR cycles was increased to 34.

### Allele Frequencies and Summary Statistics

We genotyped 103 Swiss unrelated individuals with the nine DIP–STR markers of Table 1. Blood and saliva samples were taken after informed consent and approval from the local ethic committee. DNA was extracted by using the QIAamp DNA Mini kit (Qiagen AG, Basel, Switzerland) according to the manufacturer's guidelines and quantified using the Quantifiler Human DNA Quantification Kit (Applied Biosystems, Zug, Switzerland). Summary statistics of population survey is reported in Table 2. Haplotype frequencies are indicated in Supp. Table S2. CEPH 1347–02 DNA was genotyped as a reference control for allele size. Its genotype is indicated at the bottom of each marker frequency data. The marker haplotypes are named according to the S or L allele at the DIP polymorphism and the allele size of the linked STR marker when this is analyzed by STR primers of Supp. Table S1. For markers MID1013-D5S490, MID1950-D20S473, and MID1107-D5S1980, the STR allele name is expressed in arbitrary numbers increasing according to the numbers of STR repeats.

**Table 2:** DIP–STR Marker Diversity

DIP–STR	Haplotype, *N*	S haplotype, *N* (frequency)	L haplotype, *N* (frequency)	Obs. Het.	*I*
MID1013–D5S490	15	6 (0.78)	9 (0.22)	0.49	0.28
MID1950–D20S473	10	5 (0.62)	5 (0.38)	0.80	0.36
MID1107–D5S1980	13	8 (0.31)	5 (0.69)	0.74	0.34
rs11277790–D10S530	15	8 (0.81)	7 (0.19)	0.73	0.26
rs60194384–D15S1514	12	7 (0.61)	5 (0.39)	0.88	0.36
rs67842608–D5S468	13	4 (0.21)	9 (0.79)	0.62	0.28
rs66679498–D2S342	11	6 (0.65)	5 (0.35)	0.69	0.35
rs10564579–D3S1282	12	7 (0.73)	5 (0.27)	0.85	0.32
rs35708668–D5S2045	19	18 (0.96)	1 (0.04)	0.85	0.07

*N*, number; Obs. Het., observed heterozygosity; *I*, probability of informative genotypes.

### Clinical Samples

Blood and saliva samples were collected under informed consent and approval from the local ethics committee, which required for the forensic sample the additional agreement of the justice office responsible of the investigation. Forensic mixed stain collected on the body of the victim and reference samples from the victim (blood) and the three suspects (saliva) were extracted with the QIAamp DNA mini kit following manufacture's instruction. PCR genotyping and electrophoresis were performed as indicated in the paragraph “Primer design and PCR conditions.” We used 4 ng of total DNA from the mixed stain to amplify DIP–STR markers. Pregnancy DNA microchimerism samples were obtained from 2 ml of maternal plasma extracted by QIAamp DNA mini kit according to manufacture's instruction. DNA was eluted in 60 µl of H_2_O, 6 µl of DNA was used for PCR. Reference samples of the mother (blood) and the father of the baby (saliva) were extracted by QIAamp DNA mini kit following manufacture's instruction. Forensic autosomal and Y chromosome STR were analyzed by using the AmpFlSTR SGM Plus PCR Amplification Kit (Applied Biosystems, Zug, Switzerland) and the Powerplex Y Amplification kit (Promega, Dubendorf, Switzerland) according to manufacturer's instruction.

### Analysis of DIP–STR Markers’ Performance

To evaluate the discrimination power of DIP–STR markers, we used real DIP–STR genotypes observed in 103 individuals to simulate in silico 5,253 pairwise DNA mixtures. For each pair of DNA, we considered both possibilities of major and minor DNA contributors for a total of 10,506 simulated DNA mixtures. Marker rs35708668-D5S2045 was eliminated because of the low information content (L haplotype frequency of 0.04). Based on these data, we counted the number of markers showing informative genotypes. The DIP–STR haplotypes of a minor DNA contributor that are nonshared with the major DNA represents the DNA profile that can be detected in the mixture. In Table 3 column 1, we summarized these results by indicating the percentage of simulated DNA mixtures showing a given minimum number of informative markers.

**Table 3:** Occurrence of Informative Markers

Empirical estimate using eight DIP–STR^a^	Expected estimate using 30 DIP–STR
Percentage of DNA mixtures (≥*N* informative markers)	
95 (≥1)	95 (≥6)
76 (≥2)	89 (≥7)
47 (≥3)	79 (≥8)
21 (≥4)	66 (≥9)
6 (≥5)	50 (≥10)

a Marker rs35708668-D5S2045 was eliminated because of the extremely low information content.

Alternatively, a theoretical estimate of the DIP–STR informativeness is also possible given the fact that the occurrence of informative genotypes depends on the presence of DIP alleles, which are unique to the minor DNA contributor. Based on Hardy–Weinberg assumptions, the probability of informative genotypes (*I*) at a given DIP–STR marker can be calculated as *I* = 2*s*^2^*l*^2^+2*s*^3^*l*+2*sl*^3^ (Fig. 1B). Where *s* and *l* are the frequencies of the S and L alleles, 2*s*^2^*l*^2^ is the probability that major and minor DNA contributors are homozygous for the opposite DIP allele (*s*^2^*l*^2^ +*l*^2^*s*^2^); whereas, 2*s*^3^*l*+2*sl*^3^ are the probabilities that the major DNA contributor is DIP homozygous either S or L and the minor DNA contributor is DIP heterozygous, respectively ([*s*^2^[2*sl*]+ *l*^2^[2*sl*]). The *I* value for the current nine DIP–STR markers is reported in Table 2. As average, our markers show a probability of being informative of 0.32, after the exclusion of the least polymorphic marker (rs35708668-D5S2045). In Table 3 column 2, we calculated the theoretical percentage of DNA mixtures with at least six to 10 informative markers by using a DIP–STR panel of 30 loci. This is based on the cumulative binomial distribution of 30 trials (markers) each one associated to a probability of being informative (success of the trial) of 0.32. Finally, we calculated the corresponding match probability of this initial set of markers. For each informative marker of a simulated DNA mixture (see above), we considered the frequency of the matching genotypes in the population. When a marker is informative of two minor alleles (Fig. 1A, “informative genotype 1”), this corresponds to the frequency of the heterozygous individual in the population. Conversely, when the marker is informative of only one minor DNA allele (Fig. 1A, “informative genotype 2”), the frequency of the matching genotype includes the frequency of the corresponding homozygous individual in addition to all of the possible heterozygous individuals who carry the observed allele together with any of the DIP opposite haplotypes. Fore each DNA mixture, the results of informative marker were multiplied under the assumption of independence of DIP–STR haplotypes and unrelated individuals. In the results section, we report a summary of the matching probability distribution calculated across 10,506 DNA mixtures.

## Results

### Principle of the Method

To circumvent the problem of the major DNA contributor masking the minor DNA of a mixture, we propose here a PCR-based method characterized by allele-specific primers capable of targeting DNA sequences, which are unique to the minor DNA. These sequences are biallelic DIP of few nucleotides, mostly between three and 15. The two possible alleles are also referred as, long allele (L) and short allele (S). Because biallelic markers have reduced information content, we propose the selection of DIP linked to STR to form a compound marker termed DIP–STR. The multiallelic haplotype composed of both DIP and STR alleles is analyzed by using PCR primers overlapping the deleted–inserted sequence on one side and downstream the STR region on the other side (Fig. 1). Studies of patterns of human linkage disequilibrium and recombination hotspots indicate that a distance of few hundred base pairs ensures the absence of recombination between DIP and STR [Gabriel et al., 2002; Reich et al., 2001]. The novelty of this marker with respect to other existing compound genetic markers (SNP–STR for example) is the use of extended sequence polymorphisms (3–15 bp of the DIP), which allow the sensitive and specific amplification of the minor DNA contributor in the presence of large quantities of foreign DNA background.

For a given DNA mixture, a DIP–STR marker can be informative or uninformative depending on the S/L allele mismatch between the two DNA to be discriminated (Fig. 1A). When major and minor DNA are opposite homozygous for the DIP allele, both DIP–STR haplotypes of the minor DNA can be targeted by allele-specific PCR. These minor DNA haplotypes may be identical or different depending on the homozygosity or heterozygosity of the linked STR (Fig. 1A, “informative genotype 1”). Instead, when major and minor DNA are homozygous and heterozygous, respectively, for the DIP alleles, the DIP–STR marker is still informative but the number of minor DNA haplotype that can be identified is only one (Fig. 1A, “informative genotype 2”). Conversely, uninformative cases arise when the minor DNA has no unique S or L allele. This occurs when major and minor DNA are homozygous for the same DIP allele or each time the major DNA is heterozygous SL.

### DIP–STR List and Haplotype Frequencies

We developed here nine DIP–STR markers characterized by DIP to STR distance ranging between 213 and 728 bp (Table 1). The deleted–inserted sequences range between 3 and 15 bp. Seven STR are di-nucleotide repeats, one tri-, and one tetra-nucleotide repeats. Six selected markers span different chromosomes and three are located on distant regions of chromosome 5 (5q11.2, 5q23.2, and 5q34). In Table 2, we report values of marker variability obtained from a survey of 103 unrelated Swiss individuals. The alleles of this compound marker correspond to the observed haplotypes formed by DIP and STR alleles. The observed heterozygosity range from 0.49 to 0.88. For the nine DIP–STR of Table 2, the number (*N*) of observed haplotypes varies between 10 and 19 and the frequencies of S versus L containing haplotypes are comparable except for marker rs35708668-D5S2045, where a single L haplotype was observed in the population surveyed.

### PCR Limit of Detection and Specificity Tests

To examine the minimal amount of DNA template required for successful amplification using S-DIP and STR primers as well as L-DIP and STR primers, we amplified serial dilutions of DNA template from individuals heterozygous for the DIP allele, regardless the STR genotype. The corresponding amounts of DNA ranged between 1 and 0.025 ng. The results across the 18 primers pairs (nine S and nine L DIP–STR amplifications) tested are the following: marker MID1013-D5S490 S/L, 0.025 ng of minimum amount of DNA template required for positive amplification; markers rs11277790-D10S530 S/L, rs60194384-D15S1514 S/L, rs10564579-D3S1282 S/L, rs35708668-D5S2045 S/L, rs67842608-D5S468 S, and rs66679498-D2S342 S, 0.1 ng of DNA template; markers MID1950-D20S473 S/L, MID1107-D5S1980 S/L, rs67842608-D5S468 L and rs66679498-D2S342 L, 0.25 ng of DNA template.

Next, we investigated the maximum DNA ratio at which is possible to amplify the minor DNA in a two-DNA mixture. To do so, we pooled different quantities of known DNA samples. For each marker, we used the most challenging informative genotypes, which occur when a minor DNA heterozygous at the DIP locus is mixed to a major DNA DIP homozygous (both SS and LL were tested) (Fig. 1A, “informative genotype 2”) irrespective of the linked STR alleles. In this case, only one minor DIP–STR haplotype can be selectively amplified. We mixed two DNA contributors at ratios 1:10, 1:20, 1:50, 1:100, and 1:1,000. We successfully amplified the minor DNA-specific haplotype either S or L for 15 PCR without a masking effect coming from the major DNA (Fig. 2A). The same DNA mixture analyzed by a standard forensic kit (NGMselect) allows detecting few minor DNA alleles at a ratio of 1:10 and 1:20. Conversely, at ratios from 1:50 to 1:1,000, no minor DNA allele can be detected (Fig. 2B). The S-primers of marker MID1013-D5S490 and rs10564579-D3S1282 produced a nonspecific amplification of the L allele of the major contributor at DNA ratios below 1:50 and 1:1,000, respectively. And the S-primers of marker rs66679498-D2S342 show a nonspecific PCR product of constant size close to the expected range of allele size. Further testing of PCR conditions including primer design will be necessary for marker multiplexing. We plan to solve the two issues of PCR specificity and one artifact during this future technical development.

### Examples of Clinical Utility and Feasibility

**Figure 3:**
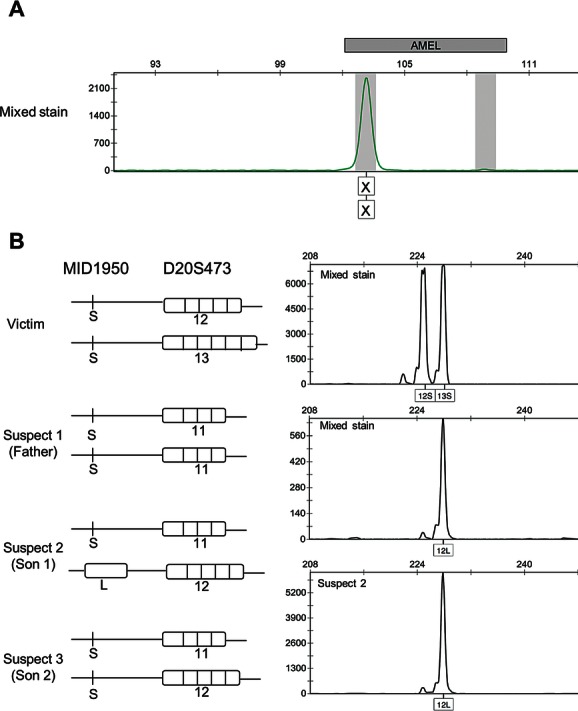
A clinical example of forensic mixed stain analysis by DIP–STR genotyping. **A:** Portion of the autosomal standard STR amplification (SGM plus kit) profile corresponding to the marker used for the sex determination, amelogenin. The unique peak indicates that no male DNA is detected in the mixed stain. The complete STR profile corresponds to the victim. The presence of a second minor DNA contributor of the mixture corresponding to a male was assessed by Y STR genotyping (data not shown). **B:** Data of one informative DIP–STR marker, MID1950-D20S473 used to discriminate between the three paternally related suspects. On the left side are the genotypes of the DNA available for comparison. On the right side, we reported the electropherograms of the mixed stain analysis when amplified by S-DIP–STR primers (upper panel) and the by L-DIP–STR primers (middle panel). The results 12S–13S of the mixed stain correspond to the DNA of the victim, while, the result 12L represents the detection of the minor DNA contributor of the mixture. Interestingly, this genotype is compatible with only one of the three suspects, suspect number 2 (lower panel).

We tested our new system on two clinical samples representative of unbalanced DNA mixtures occurring in forensic casework and peripheral blood DNA microchimerism. The forensic case concerned a woman homicide where circumstantial evidence indicated three suspects; a man and his two sons from different mothers. This setting allowed us to compare our method to the two most employed forensic analytical approaches, that is, autosomal STR and Y chromosome STR. The samples available included a biological stain collected on the body of the victim and the DNA of three suspects (Fig. 3). We first amplified 1 ng of total DNA obtained from the mixed stain by using standard autosomal STR multiplex kit. In Figure 3A, we report the mixed stain DNA profile for the amelogenin genotype, a forensic marker commonly used to determine the sex of the DNA donor. This analysis showed one autosomal STR profile, corresponding to the victim with no indication for the presence of a male DNA minor contributor (second bin in the electropherogram). However, when we specifically searched for the presence of male DNA in the stain sample by Y STR markers, we could amplify one Y chromosome profile corresponding to the three paternally related suspects. These results show that the quantity of DNA of the victim is large enough to mask the minor DNA contributor for standard autosomal loci. Since the victim was a woman, this minor fraction can be targeted by Y STR, however in this case the suspects are a father and his two sons. Therefore, the Y profile does not allow discriminating between them. To solve this issue, we genotyped some of the DIP–STR markers of Table 1. In Figure 3B, we report one informative marker, MID1950-D20S473 as a representative example. When we amplified 4 ng of DNA extracted from the mixed stain with S-DIP–STR primers, we detected the haplotypes 12S–13S, which correspond to the victim (major DNA fraction). Conversely, when we used the L-DIP–STR primers, we detected the haplotype 12L, which corresponds to the minor DNA contributor of the mixture. Interestingly, this haplotype matches only one of the three suspects, suspect number two and excluded the two others. For this specific case report, the DNA quantity selected for DIP–STR amplification was decided on the basis of the DNA required for positive Y chromosome amplification. The development of a specific DIP–STR quantification scheme for forensic applications could be useful.

**Figure 4:**
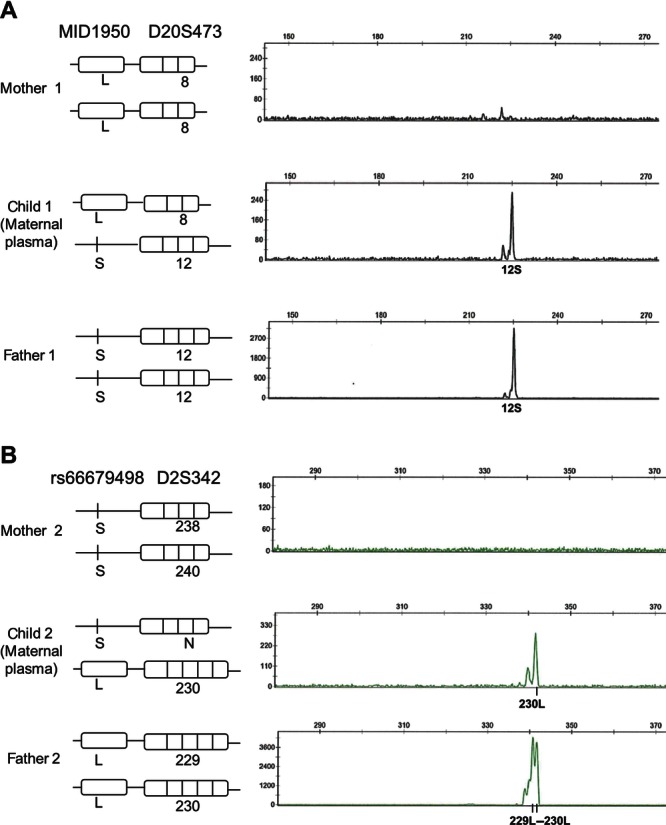
Clinical examples of pregnancy induced DNA microchimerism analysis by DIP–STR genotyping. **A:** DIP–STR amplification of marker MID1950-D20S473 using the S-specific primer on the trio, mother 1 (saliva), child 1 (maternal plasma sample drawn at 30 weeks of pregnancy) and father 1 (saliva). **B:** DIP–STR amplification of marker rs66679498-D2S342 using the L-specific primer on a different trio, mother 2 (saliva), child 2 (maternal plasma sample drawn at 36 weeks of pregnancy), and father 2 (saliva). The electropherograms show the detection in the maternal blood of the DIP–STR haplotypes transmitted from each father to the child. N; not determined.

The peripheral blood DNA microchimerism examples comprise two plasma samples from women at 30 and 36 weeks of pregnancy (Fig. 4). Knowing that the two developing babies were males, we used the Y STR markers for resolving the DNA mixture (data not shown). We further analyzed these samples by using DIP–STR markers and report here two informative results. The first plasma sample was analyzed by marker MID1950-D20S473 by using S-DIP–STR primers (Fig. 4A). The second plasma sample was analyzed by marker rs66679498-D2S342 (Fig. 4B) by using L-DIP–STR primers. In both plasma samples, we found that DIP–STR genotyping did detect the autosomal fetal haplotype transmitted from the father to the child. While this shows that our system has equal sensitivity than Y-STR for males, its principal advantage is that based on targeting autosomal sequences, it enables the analysis of fetal DNA for any sex of the baby.

### Analysis of DIP–STR Markers’ Performance

Although we tested with success our DIP–STR method on representative clinical samples, a formal evaluation of the performance of the developed DIP–STR markers is mandatory. Here, we empirically estimated the probability of finding informative markers on DNA mixtures simulated by using real DIP–STR genotypes observed in 103 Swiss individuals (marker rs35708668-D5S2045 was eliminated because of low information content, see *Methods*). Over the analysis of 10,506 in silico simulated pairwise DNA mixtures, we found that 5% of the mixtures have zero informative markers, 95% have at least one informative marker, 76% have at least two informative markers, and 47% at least three informative markers (Table 3 column 1).

Since the chances of having informative and uninformative markers depend on the occurrence of DIP alleles unique to the minor DNA, we can calculate this probability (*I*) by the function: *I* = 2*s*^2^*l*^2^+2*s*^3^*l*+2*sl*^3^ (see *Methods* and Fig. 1B). This function has a maximum at *s* = *l* = 0.5 where *I* = 0.375. The curve slowly decrease, at minor allele frequency (MAF) = 0.4 *I* = 0.365, and MAF = 0.3 *I* = 0.332. This means that given similar allele frequencies of S and L alleles, roughly one third of the cases will show an informative genotype. The *I* value for the current nine DIP–STR markers is reported in Table 2. As average our markers show a probability of being informative of 0.32, after the exclusion of the least polymorphic marker. This value can be used to determining the percentage of DNA mixtures showing a given minimum number of informative markers with respect to the size of the DIP–STR panel used (see *Methods*). In Table 3 column 2, we calculated this percentage assuming the use of 30 DIP–STR markers of allele frequencies similar to the ones already developed (*I* = 0.32). The results indicate 95% of DNA mixtures with at least six informative markers, 79% with at least eight informative markers, and 50% with at least 10 informative markers. Finally, for the eight markers described, we calculated the expectation on match probabilities based on the simulated DNA mixture data. The index of match probability is used mainly in forensics to express the probability of a random match between two DNA. The simplest estimate of the random match probability expected for an observed genotype corresponds to the frequency of the genotype in the population. In our estimates, we took into account the fact that DIP–STR can be fully (Fig. 1A, “informative genotype 1”) or partially informative (Fig. 1A, “informative genotype 2”) and therefore one frequency or multiple frequencies corresponding to all possible compatible genotypes were considered (see *Methods*). We found that 20% of simulated DNA mixtures show a minor DNA match probability between 1 and 1/10; 25% between 1/11 and 1/100; 24% between 1/101 and 1/1,000; 16% between 1/1,001 and 1/10,000 and 15% more than 1/10,000. (A more detailed analysis of the forensic value of the current DIP–STR will be the topic of a future article). Taken together, we provide examples of eight highly sensitive, specific, and informative DIP–STR markers for unbalanced DNA mixture analysis.

## Discussion

In this study, we propose the DIP–STR as a new molecular marker for the analysis of unbalanced DNA mixtures. This new tool is characterized by the following key features: (1) high sensitivity for detecting the minor DNA component of a mixture (DNA ratio 1:1,000) under informative genotypes; (2) capacity of targeting the minor DNA regardless of the sex of the contributors; (3) high discrimination power for identity testing; finally (4) simple and low-cost genotyping technique. The attractive features of this type of genetic marker reside on its compound nature: the 3–15 bp sequence polymorphisms (DIP) located close to the STR allows designing highly specific PCR primers that can target in a robust way the amplification of a minor DNA contributing less than 0.1% to a mixture.

Applications of this method include the analysis of mixed stains for forensic purposes as well as the analysis of peripheral blood DNA microchimerisms during pregnancy and following organ transplant. Our results suggest that both types of DNA mixtures can be resolved by DIP–STR analysis. In the reported clinical examples, we show that standard molecular methods either fail (autosomal STR) or provide data that are less informative (Y STR). For example, in the forensic case report, we were able to detect the presence of a minor DNA contributor of a mixed stain while autosomal STR failed. Additionally, we successfully discriminated among the three potential contributors of the minor DNA (suspects of the crime), while these cases could not be resolved by Y STR analysis, because paternally related. The expectation on match probabilities of this initial set of DIP–STR roughly corresponds to the values one can expect by using Y STR markers. Although these markers are capable of amplifying a minor DNA that is otherwise loss under major alleles, stutters, and background noise, a larger set of DIP–STR is required to obtain match probabilities comparable to autosomal STR profiling.

The suitability of the method for peripheral blood DNA microchimerism detection was demonstrated by the analysis of two plasma samples drawn from women at 30 and 36 weeks of pregnancy. In these sample analysis, our markers performed as well as the Y STR with the advantage of being able to detect both male and female developing babies. The last clinical examples confirmed the performance of our molecular assay on in vivo DNA mixtures. However, the contribution of our method to prenatal genetics will be assessable once samples from early gestational age (8–10 weeks) are included, as this stage is the most relevant for a better handling of the diagnosis of a genetic condition. In this context, previous reports indicate that the DNA microchimerism generated in the maternal blood during pregnancy is due to fetal cell turnover events occurring during the formation of the placenta [Lo et al., 1998; Tjoa et al., 2006]. The estimated quantity of fetal DNA varies between 3% and 6% of the total cell-free DNA in maternal circulation during early and late pregnancy [Lo et al., 1998]. Based on these data, we expect that DIP–STR will detect the DNA of the baby even in early pregnancy despite the slight reduction of the expected DNA quantity. Analytical methods capable of detecting the fetal DNA circulating in maternal blood can be used for developing prenatal tests including sex determination, paternity tests [Guo et al., 2012] (largely demanded in case of pregnancy after a sexual assault) and possibly diagnostic tests for specific genetic diseases. Besides allowing an earlier prenatal diagnosis, the use of noninvasive techniques eliminates the risk of fetal miscarriage associated to amniocentesis or chorionic villus sampling.

Similarly, in solid organ transplant DIP–STR development may identify noninvasive transplant surveillance biomarkers. When an individual is transplanted of an organ, shortly after the operation, traces of DNA of the donor can be detected in the blood of the recipient (and in urine for kidney transplantation) [Chok et al., 2002]. Several studies suggest that this phenomenon is caused by apoptotic events of specific cell types within the allograft. Therefore, the detection and longitudinal quantification of the donor's DNA in the blood of the recipient may correlate to the incidence of acute rejection [Gadi et al., 2006; Moreira et al., 2009; Pujal and Gallardo, 2008]. The relevance of the contribution of DIP–STR to this field resides on the fact that current methods for the diagnosis of graft injury still require invasive biopsies, and detectable pathological changes can be associated to advanced stages of allograft damage. For purposes of transplant monitoring, the quantitative feature of the assay rather than the identification power becomes relevant (the donor's alleles are known). In this respect, real-time PCR can be used to quantity of donor's DNA per milliliter of plasma. To compare several measures at different time points, the efficiency of the DNA extraction process needs to be normalized. To do so, an exogenous DNA can be spiked into the plasma, next it can be extracted and amplified together with the extracellular DNA. Several synthetic DNA exist on the market, of various lengths, provided of real time PCR primers.

Although we provide a proof of principle study, including an initial set of novel genetic markers, their applications to two clinical examples and performance studies; in practice, the contribution of DIP–STR to each field will depend on the further development of additional markers characterized by the following features: first, the DIP to STR distance should be about 200–300 bp to assure the feasibility of DNA analysis of degraded samples and of extracellular DNA generated by necrosis and apoptotic events; second, markers should be located on different chromosomes or different chromosomal arms to ensure independence of transmission for a maximum probative value; tri-, tetra-, penta-, hexa-nucleotide DIP-linked STR should be preferred to di-nucleotide STR because of the reduced problem of PCR stutters; fourth, for forensic applications, DIP–STR markers should be based on large population survey and family trios to select them universal and outside recombination hot-spots; fifth, for prenatal disease diagnosis, genetic markers can be used to determine the paternal transmission of a mutation responsible in one or two copies of a single-gene disorder. To do this, selected DIP–STR should avoid recombination hotspots to allow tracking the paternal transmission with certainties; however, these markers do not need to be linked to the disease mutation in the general population. Finally, the appropriate statistical frame should be developed for each application (the forensic statistical frame for the analysis of DIP–STR is the topic of a submitted research article).

With the aim of developing a proof-of-concept panel, here we show a limited set of markers of various features to prove the molecular feasibility of the method and provide the theoretical basis and guidelines for new marker development. It should be noted that the markers described were searched among well-characterized STR polymorphisms genotyped in several populations (7,469 STR) and DIP polymorphisms were obtained from a previous track (SNP131) of the variations and repeats UCSC database. These criteria resulted in an undersized list of candidate DIP–STR in the genome (70 markers). However, the initial analysis of the draft sequence of the human genome concluded that more than one million STR may be present depending on how they are counted [Ellegren, 2004] with 10,000 commonly used for genetic linkage and association studies. The discovery of DIP is less advanced; however, Mills and colleagues [Mills et al., 2006, 2011] described 1.36 million DIP in the genome, the majority smaller than 100 bp insertion–deletion, with an average distance location of 1.6 kb. Therefore, these studies support an a priori chance of finding enough DIP-linked STR that satisfies most of the aforementioned selecting criteria. To give a better feel of the likelihood of finding additional DIP–STR in the genome, we further explored the UCSC genome database and we searched for candidate DIP and STR polymorphisms matching the above-discussed guidelines. We search only over noncoding regions, far at least 500 bp a 3′end and 2,000 bp a 5′end of a coding sequence; simple repeats of unit length between three and six nucleotides with at least 10 repetitions to increase the chance of polymorphic STR. DIP were searched among polymorphisms annotated as indels, deletion, or insertions of 3–10 bp. Before assessing the linked markers between these two lists, we eliminated those indels that are also labeled as simple repeats and we merged redundant simple repeats annotations corresponding to the same repetitive sequence region. Based on these selecting criteria, we found 2,570 candidate DIP–STR between 25 and 200 bp apart, and 6,533 up to 500 bp apart, spanning evenly the genome from chromosomes 1 to 22. Their density should allow choosing a set of variable DIP–STR markers unlinked to each other and far from recombination hotspots. Conversely, available DIP databases currently do not offer the choice of DIP linked to consensus STR (ENFSI/CODIS loci) to allow database DNA profile search and cold-case resolution. Thus, a special effort should be put in defining new forensic databases, which include DIP–STR genotypes.

A caveat of this method that merits an extensive discussion is the risk of having DIP–STR genotypes, which are uninformative for mixture resolution. More specifically, the DIP–STR target amplification of the minor DNA is limited to those haplotypes that contain DIP alleles, which are not shared with the major DNA. It follows that, when the DIP genotype is uninformative (all minor DNA DIP alleles are shared with the major DNA), the resolution of the technique is the same as for standard STR markers. This limit is compensated by the use of a relatively large panel of DIP–STR, which increases the chance of finding alleles unique to the minor DNA, in a range that is useful in practice. It should be noted that DIP–STR markers may be advantageous over standard STR analysis even for the analysis of mixtures of two DNA in equal proportions. This is because DIP–STR allow attributing to each DNA fraction the STR alleles that are shared between the two contributors, given that they can be targeted by S- or L-specific amplifications. The question is now, how large should be an analytical DIP–STR panel? Under similar S and L allele frequencies, the minor DNA contributor is expected to have one or two detectable haplotypes (informative markers) in about one third of the genotyped markers (see *Results*). From here, we can extrapolate that the use of 30 markers characterized by a probability of being informative of 0.32 (similar to the eight preliminary DIP–STR developed), we obtain 95% of DNA mixtures with at least six informative markers and 50% of DNA mixtures with at least 10 informative markers. The precise number of markers to be genotyped will depend on the degree of polymorphism of the selected analytical panel and on the purpose of the DNA analysis.

Another foreseen valuable forensic application of the method is the screening of a complex DNA mixture for the presence of an individual following a mass disaster (DVI cases). The design of a very large panel of DIP–STR composed of rare DIP alleles can be used for searching a minor DNA in a complex DNA mixture. A similar issue by using rare SNPs was evaluated and developed theoretically [Voskoboinik and Darvasi, 2011]. In this respect, DIP–STR offer the advantage of being capable of detecting the presence of an individual in a complex mixture even when its contribution is less than 0.1% in quantity.

Finally, DIP–STR may also impact studies on human demography and population structure. This is because nonrecombining haplotypes composed of both slow-evolving markers (DIP) and fast-evolving markers (STR) combine the benefits of both tools [de Knijff, 2000; Mountain et al., 2002]. Described in simple terms, the instability of the STRs results in formation of many alleles proportional to the population divergence time and the stable flanking DIP marker allows greater certainty in tracing the lineage of each haplotype. As opposite to forensic panel, markers informative of population history show large allele frequency differences across populations including alleles, which are specific of a demographic group.

## Conclusions

DIP–STR should provide forensic scientists with a powerful tool for the investigation of a particular and yet frequent type of sample that nowadays remains unsolved. The standard technical requirements for the molecular analysis of a DIP–STR, and the planned theoretical development, will allow readily introduction of the method to forensic laboratories; however, entirely new or additional DIP–STR markers will need to be developed for each specific applications. Moreover, this new tool should allow the development of a powerful noninvasive methodological approach to tackle emerging medical questions such as, prenatal paternity tests, organ transplant monitoring, and prenatal disease diagnosis.

## Supplementary material

Disclaimer: Supplementary materials have been peer-reviewed but not copyedited.
