# Supplementary material for: DIP–STR: Highly Sensitive Markers for the Analysis of Unbalanced Genomic Mixtures
Source: Hum Mutat. 2013 Jan 25;34(4):644–54. doi: 10.1002/humu.22280 (PMC3675636; doi:10.1002/humu.22280)
Supplement: Supplementary file 1 [file humu0034-0644-SD1.pdf]

Supp. Table S1. DIP-STR primers

Marker	DIP primers	STR primers	S- and L-DIP allele specific primers
MID1013-D5S490	NED-AGGATCTCATGCAGGATAC<br>TGTTGTTTAGCTTCCTGGAC	VIC- GAATCTGAAGGTGTTCTAAAAGTAA<br>*AAAGTGAGGAGTCAAGGAGG	S-6FAM-GGTCTGTCATTACCCA.GTATTC<br>L-6FAM-GGTCTGTCATTACCCA.CTGG
MID1950-D20S473	6FAM- AAAGTGTGTCAGATCATTTGAA<br>ATCCTTCTGGAAAGATGCTT	*NED- TCATGAGCTAAATATTACTCAGTGC<br>CTTATAGCTTTTTTCAAATGATCTG	S-AAAGATGCTTTATATTTCCAGTT.TAG<br>L-AAAGATGCTTTATATTTCCAGTT.ATT
MID1107-D5S1980	VIC-CTGAAGCTAAGAAATGCTAAAAA<br>GGCACCTTAGTGATATGTGG	*NED- CATGTTTGTCACCTAGTAAAGACC<br>ATGAAATGTACCTGCCTTTG	S-TTTACTACTAGGAGGCTCTCTT.CATC<br>L-TTTACTACTAGGAGGCTCTCTT.TGTT
rs11277790-D10S530	VIC-GCTCAAAGATAAGGCCAGGA<br>TGTGTGTTTTCCCAAGTTCC	*6FAM-TCTAGCAGTAAGAGTTGTGTCTCC<br>TTGACAAGGCCATCAAAAC	S-TGAATCCTTTGCTGTAATCTC.GGGA<br>L-AATCCTTTGCTGTAATCTC.TCCAACT
rs60194384-D15S1514	VIC-CAGCATGACATTAAATCAAAAAGAA<br>TTGTGACAATGAAATTCTTGGTTT	AATTGGAAATATACGTATAAACTCA<br>*6FAM-GTCACTGCCACTGGGATATT	S-AGAGAGAATCAAAAGTCAA.ATATAC<br>L-AGAGAGAATCAAAAGTCAA.TCTTAA
rs67842608-D5S468	VIC-CAGATGTTAGCACTGGTTTGC<br>TTTTCTGTGCTTGCCTCTCA	*6FAM-AAATGAATGGTAGATTTAACCTGAG<br>TGGGAAAATAAATACATGCG	S-TCATGATCCTTAAAAGT.TTTTGG<br>L-TCATGATCCTTAAAAGT.TGGTTTAA
rs66679498-D2S342	VIC-CAATTGTTGAATGGTTTTGTTGA<br>TTTTGAAGGATGCTGTGGTG	*VIC-CTGAGAGTCTGCACTGG<br>CTCAACAAAACCATTCAACA	S-AAAGAAGACACTATTGT.GGGG<br>L-AGACACTATTGT.GTAGGAGAA
rs10564579-D3S1282	VIC-CCCAAGTCACTGTCCATCAA<br>TTTGTAGGGCTTGGAAGAAAA	*VIC-GGCAGTACCACCTGTAGAAATG<br>GAGTAACAGAGGCATCGTGTATTC	S-CTCCTACCTTGAAAAAGACTT.TATAATG<br>L-CTCCTACCTTGAAAAAGACTT.TATGAC
rs35708668-D5S2045	VIC-AACCTGTCTTGGGGCTTACA<br>ACTTGTCACCCCACTTGTCC	CAGAGACAGTCATTTCACAGAAGAG<br>*VIC-CAGCCCCATACTTGCTTG	S-CATTACTAGGGTGAC.CCAG<br>L-ACTAGGGTGAC.TACTATGTAC

* Primer used for DIP-STR amplification with S- or L-DIP allele specific primer. In the last column dots indicate the insertion/deletion points.

Supp. Table S2. DIP-STR haplotype frequencies

MID1013-D5S490		MID1950-D20S473		MID1107-D5S1980		rs11277790-D10S530		rs60194384-D15S1514		rs67842608-D5S468		rs66679498-D2S342		rs10564579-D3S1282		rs35708668-D5S2045	
S11	0.005	S8	0.005	S9	0.005	S191	0.010	S112	0.010	S88	0.005	S232	0.005	S144	0.020	S200	0.010
S12	0.005	S10	0.005	S16	0.005	S193	0.524	S120	0.020	S90	0.010	S234	0.505	S146	0.094	S202	0.029
S13	0.034	S11	0.267	S17	0.005	S195	0.097	S124	0.206	S92	0.097	S236	0.015	S148	0.020	S204	0.397
S14	0.694	S12	0.286	S18	0.005	S197	0.010	S128	0.235	S94	0.102	S238	0.069	S150	0.040	S206	0.054
S15	0.034	S13	0.053	S19	0.203	S199	0.102	S132	0.098	L86	0.039	S240	0.054	S152	0.163	S208	0.010
S16	0.005	L8	0.073	S20	0.054	S201	0.029	S136	0.039	L88	0.005	S247	0.005	S154	0.376	S211	0.010
L15	0.015	L9	0.005	S21	0.030	S203	0.024	S140	0.005	L90	0.073	L228	0.020	S158	0.015	S213	0.093
L20	0.053	L11	0.019	S22	0.005	S205	0.010	L120	0.025	L92	0.034	L229	0.137	L146	0.114	S214	0.005
L21	0.029	L12	0.184	L13	0.465	L179	0.010	L124	0.078	L94	0.553	L230	0.167	L148	0.005	S215	0.083
L22	0.015	L13	0.102	L14	0.050	L181	0.005	L128	0.147	L96	0.039	L245	0.020	L150	0.119	S217	0.074
L23	0.073			L15	0.054	L183	0.015	L132	0.093	L98	0.034	L247	0.005	L154	0.025	S218	0.010
L24	0.019			L17	0.109	L191	0.010	L136	0.044	L100	0.005			L156	0.010	S219	0.108
L25	0.005			L19	0.010	L197	0.063			L102	0.005					S221	0.025
L26	0.010					L199	0.078									S223	0.025
L27	0.005					L203	0.015									S225	0.010
																S227	0.005
																S229	0.010
																S231	0.005
CEPH1347-02 DNA																L198	0.039
S13		S11		S20		S193		S124		S92		S234		S154		S213	
S14		S11		L13		S205		S128		L94		S238		S156		S215	
